# Supplementary material for: Clinicopathological Features and Survival Outcomes of Primary Pulmonary Invasive Mucinous Adenocarcinoma
Source: Cancers (Basel). 2021 Aug 15;13(16):4103. doi: 10.3390/cancers13164103 (PMC8393953; doi:10.3390/cancers13164103)
Supplement: Supplementary file 1 [file cancers-13-04103-s001.zip › cancers-1325413-supplementary.pdf]

Supplementary Materials and Methods

Table S1. Primers used for *NRG* fusions and *HER2* transmembrane domain.

**Gene**	**Forward**	**Reverse**
SLC3A2-NRG1 fusion	5 ´ - CTGACTTGCTGCAGATCGAC -3 ´	5 ´ - ATCTCGAGGGGTTTGAAAGG -3 ´
VAMP2-NRG1 fusion	5 ´ - GCCATCATCCTCATCATCATC -3 ´	5 ´ - ATCTCGAGGGGTTTGAAAGG -3 ´
HER2 transmembrane domain (TMD)	5 ´ - CGGTGTGAAACCTGACCTCT -3 ´	5 ´ - CAGCTCCGTCTCTTTCAGGA -3 ´

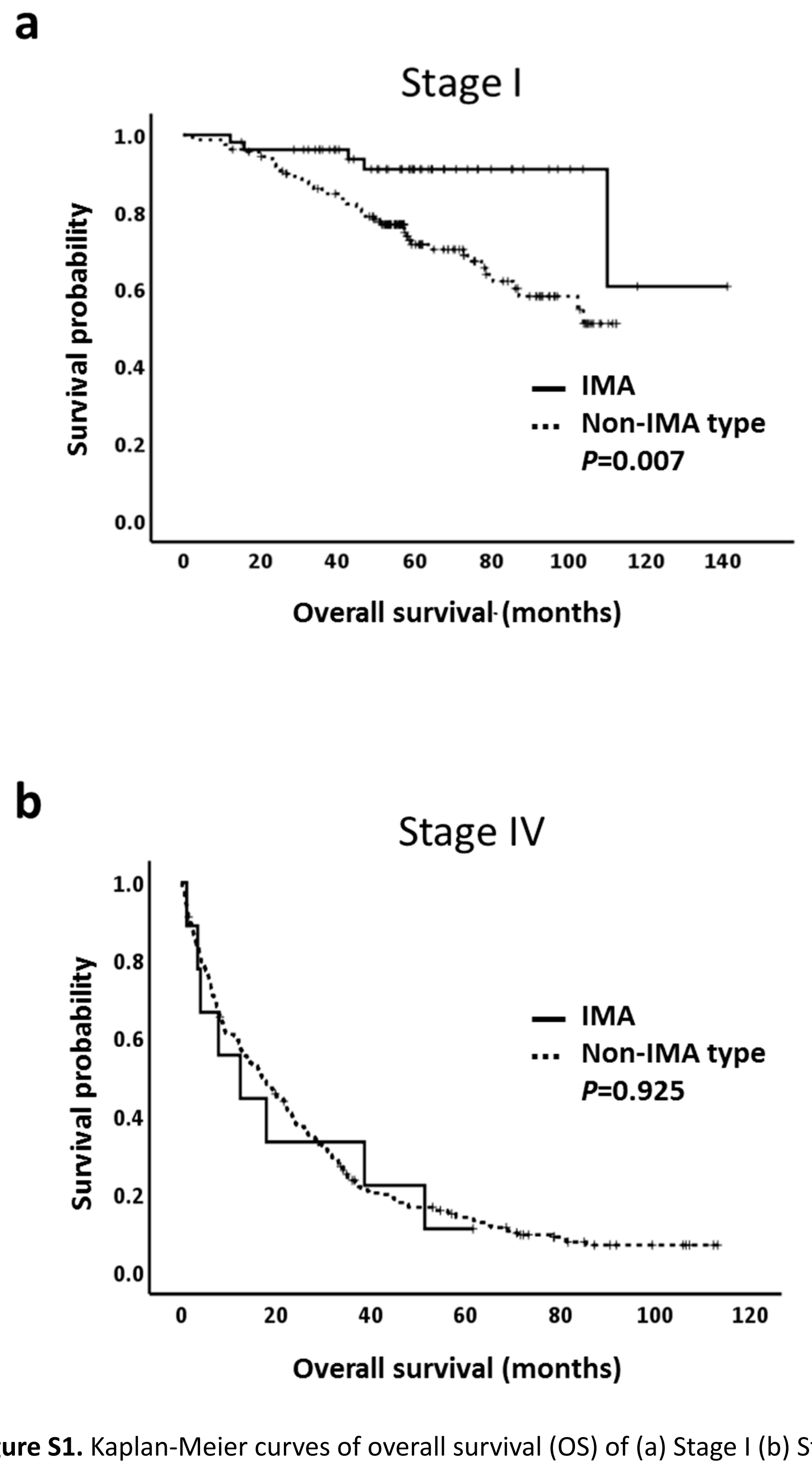

Supplementary Figure S1. Kaplan-Meier curves of overall survival (OS) of (a) Stage I (b) Stage IV patients with pulmonary invasive mucinous adenocarcinoma and non-IMA type adenocarcinoma.
